# Intestinal Parasites in First World War German Soldiers from “Kilianstollen”, Carspach, France

**DOI:** 10.1371/journal.pone.0109543

**Published:** 2014-10-15

**Authors:** Matthieu Le Bailly, Michaël Landolt, Leslie Mauchamp, Benjamin Dufour

**Affiliations:** 1 University of Franche-Comte, CNRS UMR 6249 Chrono-Environment, Besancon, France; 2 PAIR, CNRS UMR 7044 Archimède, ZA Sud, CIRSUD, Sélestat, France; Universidade Federal de Minas Gerais, Brazil

## Abstract

Paleoparasitological investigations revealed the presence of intestinal helminths in samples taken from the abdominal cavities of two German soldiers, recovered in the First World War site named “Kilianstollen” in Carspach, France. Eggs from roundworm, whipworm, tapeworm and capillariids were identified. The morphological and morphometrical comparison, followed by statistical analyses, showed that the Carspach capillariid eggs are similar to rodent parasites. Poor sanitary conditions in the trenches, the lack of knowledge of parasites, and the widespread presence of commensal animals, can explain the occurrence of such parasites in human intestines. This study is the second dealing with 20^th^ century human samples. It confirms the presence of intestinal worms in First World War German soldiers. In this case study, the application of statistics to precise measurements facilitated the diagnosis of ancient helminth eggs and completed the microscopic approach.

## Introduction

Paleoparasitology has developed over the past century and is recognized as a branch of parasitology specialized in studying ancient parasites recovered from paleontological and archaeological sites [1], [2]. As this research field develops, it increasingly uses modern tools to recover and identify parasites, thereby pushing back the frontiers of the discipline in terms of time scale and possibilities. Although different parasite residues can be recovered in archaeological samples, i.e., macro-remains, dissemination forms or biomolecules, intestinal helminth eggs have been the most widely studied markers since the early stages of paleoparasitology. However the diagnosis of parasite eggs under microscopy remains difficult as egg characteristics (morphology and morphometry) do not often lead to a specific diagnosis, but also because these characteristics disappear over time as a result of taphonomy. In the 1980s, researchers interested in the study of ancient parasites began applying statistical approaches to attempt to differentiate ancient eggs. This was the case for whipworm eggs (*Trichuris* sp.), one of the most prevalent parasites in archaeological sites [3], [4]. More recently, paleoparasitologists used statistical approaches in order to identify capillariid eggs recovered in various contexts in Argentina, and confirmed the efficiency of such a tool for paleoparasitological diagnoses [5], [6], [7]. Here, we present the results of an original study carried out on modern samples, dated to the First World War, using statistics to complete the classical paleoparasitological analyses and to improve the diagnosis of intestinal parasite eggs.

## Materials and Methods

### Site presentation

**Figure 1:**
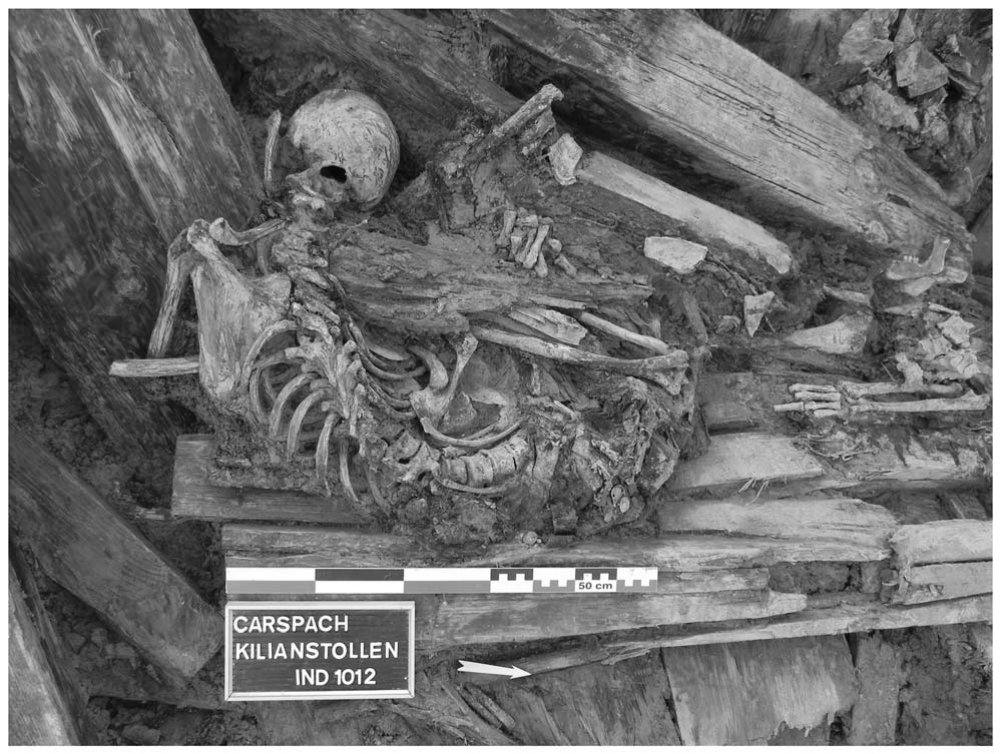
The soldiers recovered during the excavations of “Kilianstollen” in Carspach. A- Soldier #1012. B- Soldiers #1018 (left) and #1019 (right) (Photo: M. Landolt).

The “Kilianstollen” was a German gallery built during the winter of 1915/1916 [8]. It was directly connected to the second trench of the first German battle line localized in Carspach (west of the city of Altkirch) in the Alsace region, France. The gallery was around 125 meters long, situated between 3.5 and 6 meters below the surface and was used as a refuge during enemy attacks. On March 18^th^ 1918, heavy French shelling on the “Kilianstollen” position forced part of the German company (94^th^ Infantry Company reserve) to seek refuge in the gallery. At around 1.30 pm, the southern part of the gallery (the shallower part) collapsed on 34 soldiers. Among them, 21 could not be saved and were left in the gallery [9]. During the excavations carried out at “Kilianstollen” in 2011, the bodies of the 21 soldiers were recovered. Sediment samples were collected from the abdominal cavity of three soldiers recovered during the excavations (Figure 1) [10], [11]. The pluridisciplinary study, involving archaeologists, anthropologists, and historians, led to the exact identification of the soldiers. Samples in our study came from a 22 years old corporal (sample #1012); a 20 years old soldier (sample #1018); and a 35 years old sergeant (sample # 1019).

### Ethic statements

Excavations of the "Kilianstollen" (47°37′51.9″N and 7°13′04.4″E) were performed by the PAIR under the direction of M. Landolt. Excavation authorization was granted by the Regional Service of Archaeology of Alsace, France (Order numbers 2007/227 and 2011/047). Three sediment samples (#1012, #1018 and #1019) were provided by M. Landolt. No authorizations were required for the sediment sample study, which complied with all relevant regulations. Samples are stored at CNRS UMR 6249, Besancon, under the responsibility of M. Le Bailly.

### Parasite extraction and microscopy

The standard RHM protocol (Rehydration-Homogenization-Microsieving) was employed to extract parasite eggs from the Carspach samples [12], [13]. Five grams of each sample were rehydrated for one week in a 0.5 % trisodic phosphate (50 ml) and 5 % glycerinated water solution (50 ml). Samples were then crushed in a mortar and immersed in an ultrasonic bath (50/60 Hz) for one minute. The homogenized samples were filtered in microsieving columns with calibrated meshes of 315 µm, 160 µm, 50 µm and 25 µm. Intestinal parasite egg size varies between 30 and 160 µm in length, and between 15 and 90 µm in width [14]. In these conditions 25 µm and 50 µm mesh residues were kept and transferred into 4 ml tubes for analyses. Observations and measurements of parasite eggs were conducted under an optical microscopy at magnifications of x100, x400 and x600 (Olympus BX-51, Saisam software by Microvision Instrument).

## Results

**Figure 2:**
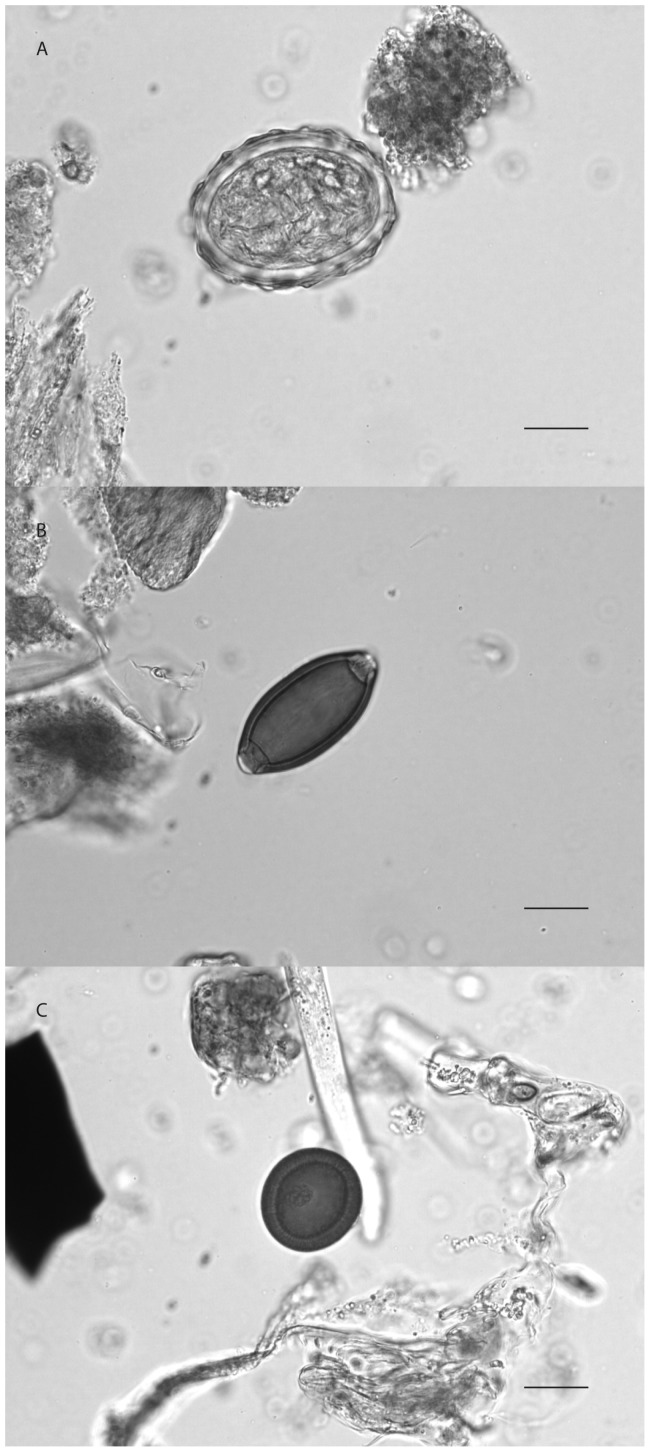
A. Egg of *Ascaris lumbricoides* (66.57×53.04 µm) recovered in individual # 1018 in Carspach “Kilianstollen”. B. Egg of *Trichuris trichiura* (53.19×27.45 µm) recovered in individual # 1019 in Carspach “Kilianstollen”. C- Egg of *Taenia* sp. (34.95×32.25 µm) recovered in individual # 1018 in Carspach “Kilianstollen”. Scale bar = 20 µm (Photos: M. Le Bailly).

Samples from body #1018 and body #1019 tested positive for intestinal helminth eggs. No parasite residue was found in samples from body #1012 (table 1). Four taxa were identified during the analyses. Human roundworm eggs (*Ascaris lumbricoides*) and human whipworm eggs (*Trichuris trichiura*) were recovered, as well as one human tapeworm egg (*Taenia* sp.), and eggs from the capillariid group. Because of the sampling context, the human origin of the samples was certain, leading to a specific diagnosis for roundworm and whipworm. Average egg sizes, respectively 73.59±4.27 µm long and 50.09±2.59 µm wide for *A. lumbricoides*, and 54.48±2.74 µm long and 27.63±0.21 µm wide for *T. trichiura*, were in accordance with species descriptions (Figures 2a and 2b). As for the tapeworm, the specific diagnosis of the recovered egg was not possible using microscopy (figure 1c) due to the fact that three different species can infect humans (*T. solium*, *T. saginata* and *T. asiatica*). Moreover, because of the size of the egg (35.18×31.94 µm), the context of world conflict and world population mixing, all species should be considered.

**Table 1:** Paleoparasitological results per sample.

Samples	*Ascaris* sp.	*Trichuris* sp.	*Capillaria* sp.	*Taenia* sp.
1012	-	-	-	-
1018	180	-	5	1
1019	-	2	1	-
Total	180	2	6	1

Values represent the number of egg after the analysis of ten slides.

**Figure 3:**
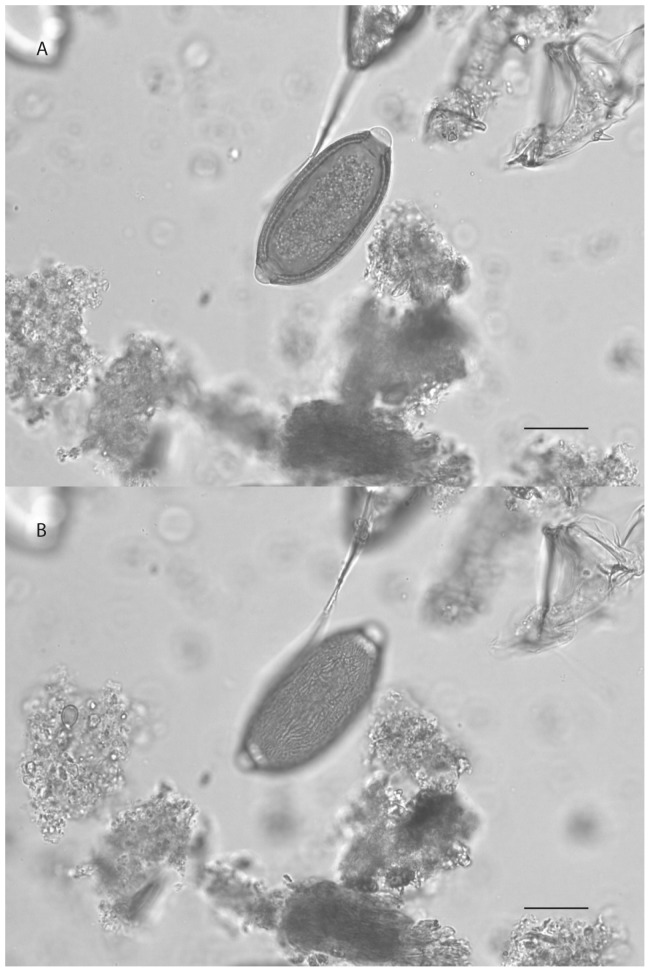
A. Egg of capillariid (65.03×28.38 µm) recovered in individual # 1018 in Carspach “Kilianstollen”. B. Details on the eggshell ornamentation showing a dense and relatively thin network. Scale bar = 20 µm (Photos: M. Le Bailly).

Capillariid taxonomy is often debated by parasitologists and the microscopic diagnosis of the eggs remains difficult. However, it was possible to propose specific hypotheses on the basis of egg descriptions using comparative and statistical approaches. Capillariid eggs recovered in the Carspach samples were barrel-shaped, with two polar plugs, symmetric sides, and presented a double-layered shell. The inner layer was hyaline, whereas the outer layer displayed fine and relatively dense network-like ornamentation. Egg measurements were 65.56±1.69 µm long (including plugs) and 28.64±0.46 µm wide (n = 6) (Figure 3).

Among the Capillariids that can infect humans, the most prevalent are Calodium hepaticum (syn. Capillaria hepatica), Eucoleus aerophilus (syn. Capillaria aerophila) and Paracapillaria philippinensis (syn. Capillaria philippinensis, Aonchotheca philippinensis) [14], [15]. Eggs of C. hepaticum are barrel-shaped with shallow polar prominences, and a distinct radiate striated shell. Egg measurements are 51–68 µm long and 30–35 µm wide [16], [17], [18], [19]. E. aerophilus eggs are barrel-shaped, asymmetric and the outer shell layer presents a thick mesh with wide depressions. Egg measurements are 59–83 µm long and 26–40 µm wide [16], [20], [21]. P. philippinensis eggs are peanut-shaped, present typical inconspicuous polar plugs and a striated outer layer. Egg measurements are 36–45 µm long and 21 µm wide [22], [23], [24]. The comparison of the egg characteristics (shape, size and ornamentation) indicated that these capillariid eggs do not correspond to those recovered in our samples. In addition, it is important to note that some species have never been mentioned in Europe, for example P. philippinensis [24], [25].

Among other mammalian capillariid species, the eggs of Capillaria putorii (syn. Aonchotheca putorii), C. bovis (syn. Aonchotheca bovis), C. erinacei (syn. Aonchotheca erinacei), Aonchotheca legerae and Eucoleus gastricus could potentially match the eggs extracted from our samples, as they present an outer shell ornamented with a network [16], [20], [26], [27], [28], [29], [30]. The network patterns on the eggs of C. putorii, C. bovis and C. erinacei look different and slacker than those of the Carspach eggs. On the other hand, Eucoleus gastricus and Aonchotheca legerae eggs, two nematodes parasitizing rodents, seem to be good possible matches.

A data set was created using all the egg criteria, which have been defined by multiple microscopic observations, and then completed with books or articles showing egg photography, as well as the egg descriptions available in the literature.

**Figure 4:**
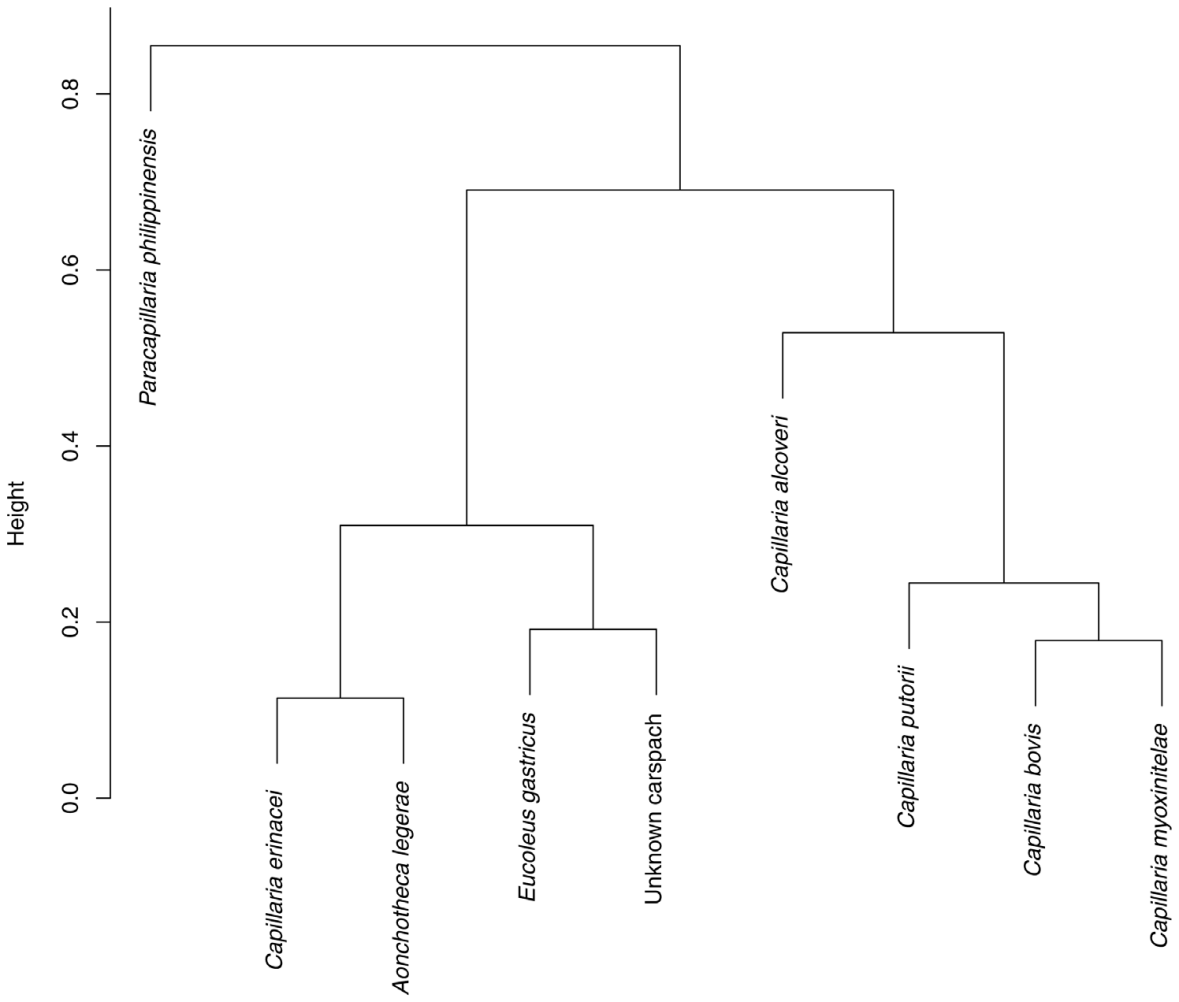
Hierarchical clustering established for capillariid reticulated eggs by using the Gower distance and the Ward minimum variance.

In order to improve the capillariid egg diagnosis, the Gower distance and then Ward's minimum variance clustering were applied to this data matrix. The latter included the morphological and morphometric criteria of eight mammalian capillariid eggs, all presenting a network-like outer shell (Table S1). The Gower coefficient was computed because of the various types of variables included in the matrix, namely qualitative (density) and quantitative variables (length, width). As visible on the h-clust dendrogram, the statistics confirmed that the Carspach eggs could be related to *Eucoleus gastricus,* a nematode parasitizing rodents (Figure 4).

## Discussion

The presence of roundworm, whipworm and tapeworm associated with humans is not new and has been identified in archaeological samples since prehistory [31], [32], [33], [34]. Moreover, in a recent study, *Ascaris* sp., *Trichuris* sp. and *Taenia* sp. eggs were recovered from German soldiers in the site of Entzheim (Alsace region, France), also dated to the First World War [35]. Their presence was probably the result of many parameters, such as bad hygiene, insalubrity, close human proximity, the poor management of organic remains, undercooked or rawfood consumption and a lack of knowledge of parasites.

The presence of capillariids in humans is much more uncommon. Capillariid eggs were recorded in South American archaeological sites in Patagonia [36], [37].In Western Europe, very few mentions of capillariids are available. The first record comes from the Neolithic sites of Chalain in France, where eggs were identified, associated with probable human coprolites [38], [39], [40]. The authors assumed that the eggs could be transmitted by wild mammals like mustelidae. The second record comes from the Neolithic cemetery of Karsdorf in Germany where a human skeleton was analyzed for intestinal parasites. Capillariid eggs presenting a network-like sculpture on the surface were identified and the authors presumed that this was due to pollution by rodent feces [41]. Finally, in the medieval sites of Namur and Raversijde, Belgium, capillariid eggs were recovered in latrines, associated with human parasites [42], [43].

In our study, the presence of capillariid eggs in human intestines also raises the question of the origin of these eggs, and the authenticity of the infection. One of the most probable hypotheses would be a case of spurious infection in which the soldiers would have accidentally ingested capillariid eggs with food, water or simply because of poor personal hygiene in the trenches. Rodents, in particular rats, which are commensal to humans, were abundant, as mentioned in soldiers' correspondence [44], [45], [46], and their feces could have caused widespread environmental pollution. Another way of contamination in the event of a spurious infection would be the direct consumption of rat meat, as attested by correspondence during the wars [47], [48]. However, the hypothesis of an actual human infection remains plausible, and would have induced a case of horizontal host transfer that cannot be proven for the time being. It is important to note that rats, *Rattus norvegicus*, were identified during the archaeozoological study in the site.

In the case of the “Kilianstollen”, the lower ranking soldier, #1018, yielded a high positivity for helminth eggs, in particular for ascariasis. The higher grades, the sergeant, #1019, and the corporal, #1012, tested slightly positive or negative. Easier access to commodities, water, and possibly a better sanitary education could explain this result.

## Conclusions

For the second time in paleoparasitology, samples dated to the 20^th^ century were studied for intestinal helminths, thereby confirming the presence of worm diseases in German soldiers during the First World conflict. Although these pathogens are ancient in Western Europe, diet and insalubrity could be the cause of or could have contributed to maintaining ascariasis, trichuriasis or tapeworm disease in soldiers, whereas the presence of infected animals, in particular rodents, surrounding humans could probably explain the case of capillariasis, as already observed during the Neolithic period [36], [38]. It remains to be established whether humans were truly infected or simply acted as a transitory host. In this study, the multiple sources of contamination may reflect the poor living conditions of the combatants and the lack of knowledge of parasitic diseases at the beginning of the last century. Studying parasites in such a modern context reminds us how parasitology knowledge is recent, but also how living conditions and parasite nosology have evolved over the past decades.

The identification of ancient parasites based on egg observation is difficult and sometimes impossible. In the case of capillariids, the comparative study of the eggshell ornamentation coupled with a statistical approach provided an appropriate solution to improve identification. Microscopy and micromorphology also appear to be a satisfactory temporary alternative to molecular analyses, which is not always applicable due to the lack of genomic data for some parasite groups. Spurred on by such tools, future paleoparasitological investigations should contribute to a better understanding of parasite cycles, history and evolution.

## Supporting Information

Table S1 **Matrix including morphological and micrometrical data of capillariid species producing eggs with network-like shell ornamentation.** Density: low = low or flack; mid = middle; den = high or dense.
(XLSX)
